# Supplementary material for: Differential but complementary roles of HIF-1α and HIF-2α in the regulation of bone homeostasis
Source: Commun Biol. 2024 Jul 23;7:892. doi: 10.1038/s42003-024-06581-z (PMC11263705; doi:10.1038/s42003-024-06581-z)
Supplement: Supplementary file 2 — Supplementary Information [file 42003_2024_6581_MOESM2_ESM.pdf]

Supplemental Information

Differential but complementary roles of HIF-1α and HIF-2α in the regulation of bone homeostasis

Sun Young Lee[1†], Su-Jin Kim[1,2†], Ka Hyon Park[1,2], Gyuseok Lee[1], Youngsoo Oh[1], Je-Hwang Ryu[1,2*], and Yun Hyun Huh[3*]

Supplementary Figure 1, 2, 3 and 4

Supplementary Table 1

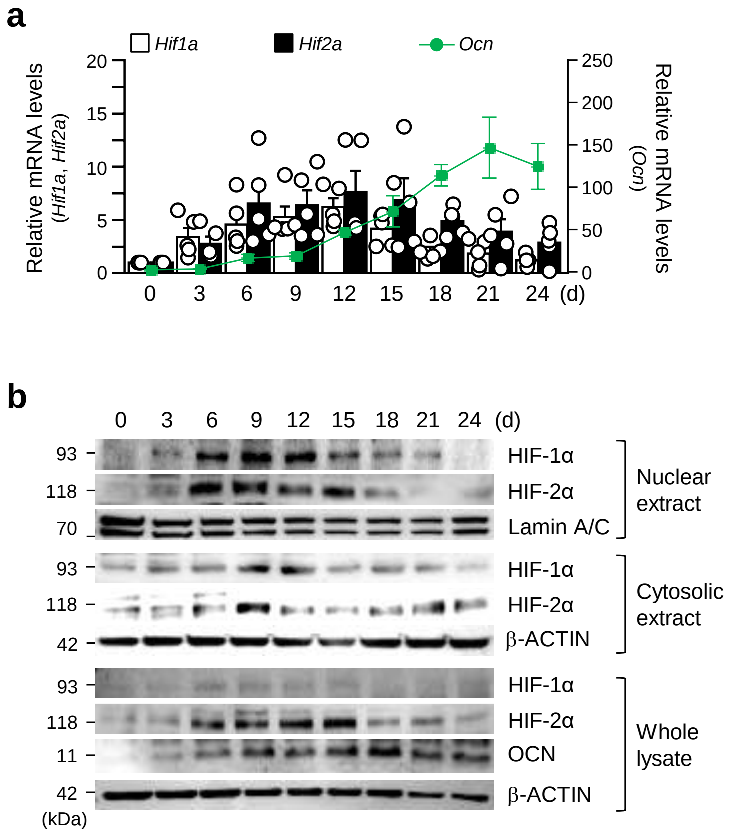

Supplementary Fig. 1 *Hif1a* is up-regulated during osteoblast differentiation. a, b Primary calvarial pre-steoblasts isolated from WT mice were cultured in the DM. Relative transcript levels of *Hif1a, Hif2a, and Ocn* at indicated culture day (0-24 days) were determined by qRT-PCR (*n* = 5) **(a)**. The protein levels and nuclear translocation were determined by western blotting (*n* = 3) **(b)**.

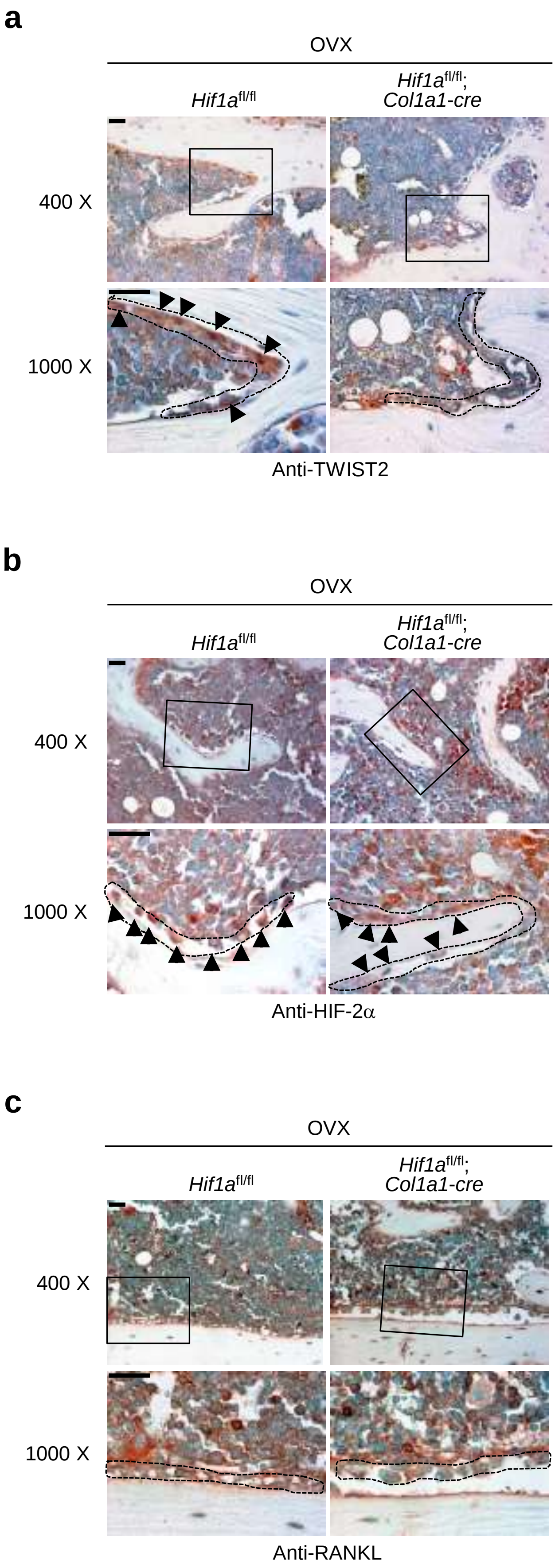

a
OVX
Hif1a^fl/fl
Hif1a^fl/fl; Col1a1-cre
400 X
1000 X
Anti-TWIST2
b
OVX
Hif1a^fl/fl
Hif1a^fl/fl; Col1a1-cre
400 X
1000 X
Anti-HIF-2α
c
OVX
Hif1a^fl/fl
Hif1a^fl/fl; Col1a1-cre
400 X
1000 X
Anti-RANKL

Supplementary Fig. 2 Representative images of immunohistochemical staining in bone tissue from OVX model in *Hif1a*$^{fl/fl}$ and *Hif1a*$^{fl/fl}$;*Col1a1-Cre* mice. a-c Representative mouse femur sections stained TWIST2 **(a)**, HIF-2a **(b)**, and RANKL **(c)**. The lower panel images are the enlarged frames of the upper panel. The dotted lines indicate osteoblasts, and the nuclear positively stained osteoblasts in brown are indicated by arrows.

Figure 2h

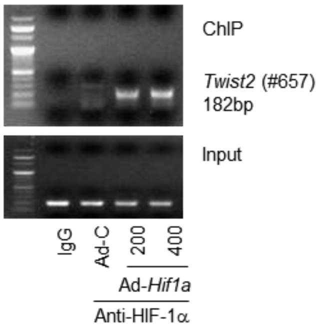

Figure 5a

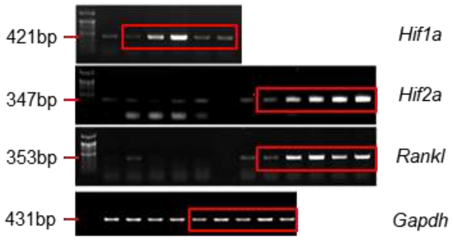

Figure 5b

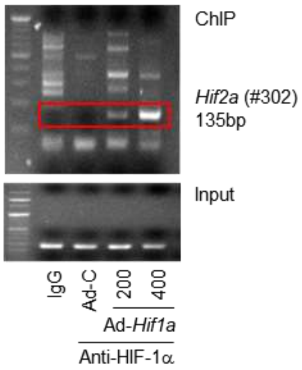

Supplementary Fig. 3 Unedited/uncropped gel for main figures.

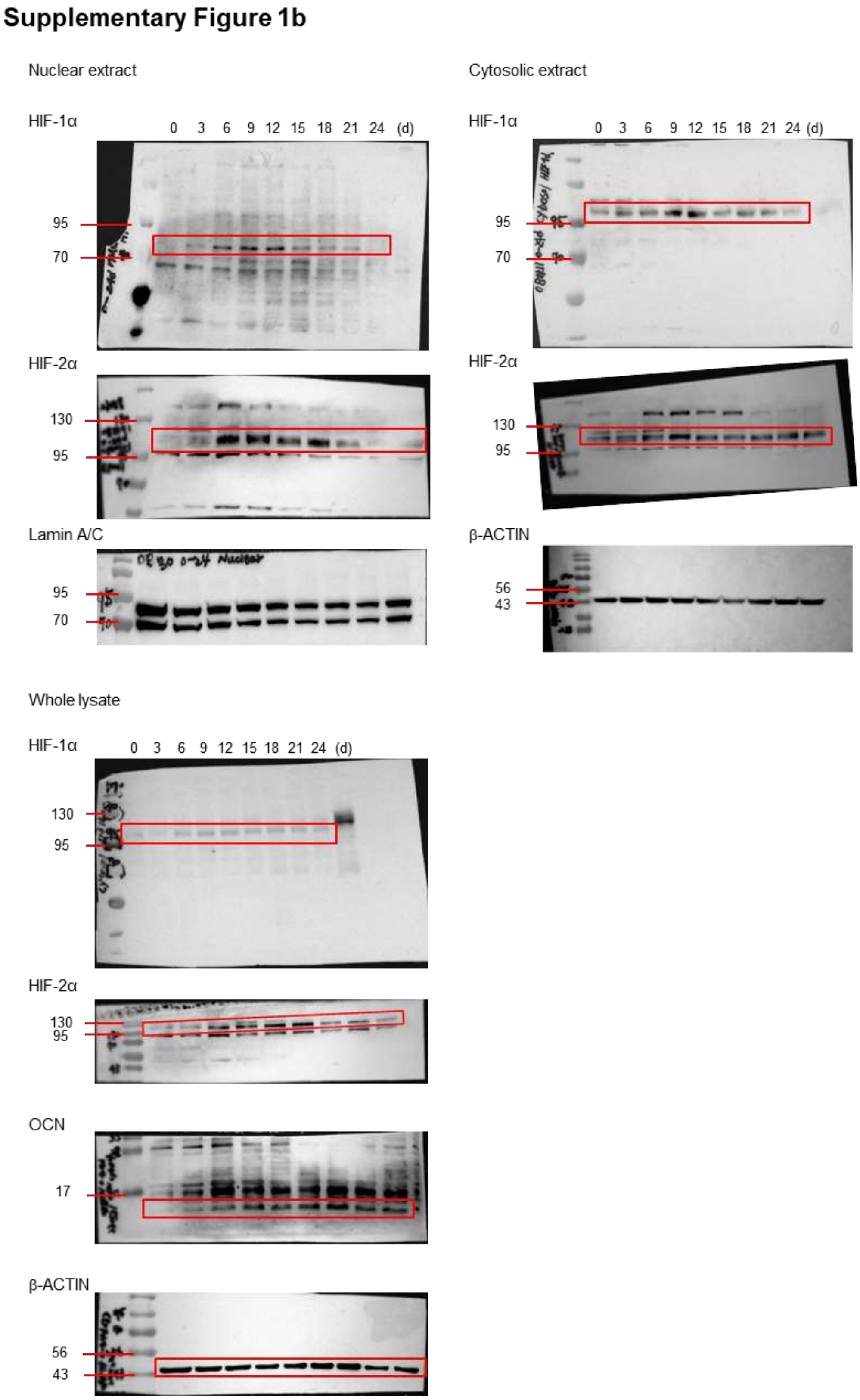

Supplementary Fig. 4 Unedited/uncropped western blots for supplementary figures.

Supplementary Table 1 PCR primers and conditions

Gene	Strand	Sequences (5’-3’)	Size (bp)	[a]AT (°C)
Alp	[b]S	TACACCACAACACGGGCGAGGAGAC	356	60
	[c]As	CCTCTGGTGGCATCTCGTTATCCG		
Hif1a	S	AGGCTCACCATCAGTTATTTACGTGTG	421	60
	As	TAGACATGAATATGGCCCGTGCAGTG		
Hif2a	S	AGAAGAGCAAAGACGTGTCCACCGAG	347	63
	As	GTAGAACTCATAGGCAGAGCGTCCAAG		
Ocn	S	CTCCTGAGAGTCTGACAAAGCCTT	320	55
	As	GCTGTGACATCCATTACTTGC		
Opg	S	CTTGCCCTGACCACTCTTATACGG	359	60
	As	CGTTGTCATGTGTTGCATTTCCTTTC		
Rankl	S	GACTCGACTCTGGAGAGTGAAGAC	353	60
	As	AATGTTGGCGTACAGGTAATAGAAG		
Runx2	S	GCCACCTTTACCTACACCCC	363	55
	As	GACTCATCCATTCTGCCGCT		
Twist1	S	TGAGCAACAGCGAGGAGGAG	417	60
	As	GCCAGTTTGAGGGTCTGAATC		
Twist2	S	GGCCGCCAGGTACATAGAC	103	60
	As	GTAGCTGAGACGCTCGAGA		
Gapdh	S	TCACTGCCACCCAGAAGA	431	60
	As	TGTAGGCCATGAGGTCCA		
Twist2 ChIP	S	CCAACACTCAGGGCAAGGTC	182	60
	As	CTCGGGCCAGTATGCAAGTTG		

Hif2a ChIP	S	AATCGCCCCCACCCCAAGAC	135	60
	As	CAATTGCGGAGAAAGCACGG		

[a]AT, annealing temperature; [b]S, sense primer; [c]As, antisense primer
