# Supplementary material for: Differential but complementary roles of HIF-1α and HIF-2α in the regulation of bone homeostasis
Source: Commun Biol. 2024 Jul 23;7:892. doi: 10.1038/s42003-024-06581-z (PMC11263705; doi:10.1038/s42003-024-06581-z)
Supplement: Supplementary file 3 — Description of Supplementary Materials [file 42003_2024_6581_MOESM3_ESM.pdf]

Description of Additional Supplementary Files

File name: Supplementary Data 1

Description: Source Data used to generate the main figures
