# Supplementary material for: Differential but complementary roles of HIF-1α and HIF-2α in the regulation of bone homeostasis
Source: Commun Biol. 2024 Jul 23;7:892. doi: 10.1038/s42003-024-06581-z (PMC11263705; doi:10.1038/s42003-024-06581-z)
Supplement: Supplementary file 5 — Reporting Summary [file 42003_2024_6581_MOESM5_ESM.pdf]

nature portfolio

Corresponding author(s): Je-Hwang Ryu

Last updated by author(s): Mar 29, 2024

Reporting Summary

Nature Portfolio wishes to improve the reproducibility of the work that we publish. This form provides structure for consistency and transparency in reporting. For further information on Nature Portfolio policies, see our Editorial Policies and the Editorial Policy Checklist.

Statistics

For all statistical analyses, confirm that the following items are present in the figure legend, table legend, main text, or Methods section.

n/a	Confirmed	
☐	☒	The exact sample size (*n*) for each experimental group/condition, given as a discrete number and unit of measurement
☐	☒	A statement on whether measurements were taken from distinct samples or whether the same sample was measured repeatedly
☐	☒	The statistical test(s) used AND whether they are one- or two-sided<br>*Only common tests should be described solely by name; describe more complex techniques in the Methods section.*
☒	☐	A description of all covariates tested
☐	☒	A description of any assumptions or corrections, such as tests of normality and adjustment for multiple comparisons
☐	☒	A full description of the statistical parameters including central tendency (e.g. means) or other basic estimates (e.g. regression coefficient) AND variation (e.g. standard deviation) or associated estimates of uncertainty (e.g. confidence intervals)
☐	☒	For null hypothesis testing, the test statistic (e.g. *F*, *t*, *r*) with confidence intervals, effect sizes, degrees of freedom and *P* value noted<br>*Give P values as exact values whenever suitable.*
☒	☐	For Bayesian analysis, information on the choice of priors and Markov chain Monte Carlo settings
☒	☐	For hierarchical and complex designs, identification of the appropriate level for tests and full reporting of outcomes
☒	☐	Estimates of effect sizes (e.g. Cohen's *d*, Pearson's *r*), indicating how they were calculated

Our web collection on statistics for biologists contains articles on many of the points above.

Software and code

Policy information about availability of computer code

Data collection	No software was used
Data analysis	GraphPad Prism version 7

For manuscripts utilizing custom algorithms or software that are central to the research but not yet described in published literature, software must be made available to editors and reviewers. We strongly encourage code deposition in a community repository (e.g. GitHub). See the Nature Portfolio guidelines for submitting code & software for further information.

Data

Policy information about availability of data

All manuscripts must include a data availability statement. This statement should provide the following information, where applicable:

- Accession codes, unique identifiers, or web links for publicly available datasets
- A description of any restrictions on data availability
- For clinical datasets or third party data, please ensure that the statement adheres to our policy

Data supporting the present study are available from the corresponding author upon reasonable request.

Research involving human participants, their data, or biological material

Policy information about studies with human participants or human data. See also policy information about sex, gender (identity/presentation), and sexual orientation and race, ethnicity and racism.

Reporting on sex and gender	N/A
Reporting on race, ethnicity, or other socially relevant groupings	N/A
Population characteristics	N/A
Recruitment	N/A
Ethics oversight	N/A

Note that full information on the approval of the study protocol must also be provided in the manuscript.

Field-specific reporting

Please select the one below that is the best fit for your research. If you are not sure, read the appropriate sections before making your selection.

☒ Life sciences ☐ Behavioural & social sciences ☐ Ecological, evolutionary & environmental sciences

For a reference copy of the document with all sections, see nature.com/documents/nr-reporting-summary-flat.pdf

Life sciences study design

All studies must disclose on these points even when the disclosure is negative.

Sample size	All data presented represent the mean ± SEM. All quantified data were first tested for conformation to a normal distribution using the Shapiro-Wilk test, followed by analysis with two-tailed Student's t-test for quantitative variables between the means of two groups or one-way ANOVA or two-way ANOVA followed by Tukey's post hoc tests (multi-comparison) for variables between the means of three or more independent groups, as appropriate. The n-value was the number of independent experiments or mice. Results were considered statistically significant when p-values were less than 0.05.
Data exclusions	No data were excluded from the analyses.
Replication	All experiments were repeated at least three times independently.
Randomization	This study did not involve a randomized trial.
Blinding	N/A

Reporting for specific materials, systems and methods

We require information from authors about some types of materials, experimental systems and methods used in many studies. Here, indicate whether each material, system or method listed is relevant to your study. If you are not sure if a list item applies to your research, read the appropriate section before selecting a response.

Materials & experimental systems

n/a	Involved in the study	
☐	☒	Antibodies
☐	☒	Eukaryotic cell lines
☒	☐	Palaeontology and archaeology
☐	☒	Animals and other organisms
☒	☐	Clinical data
☒	☐	Dual use research of concern
☒	☐	Plants

Methods

n/a	Involved in the study	
☒	☐	ChIP-seq
☒	☐	Flow cytometry
☒	☐	MRI-based neuroimaging

Antibodies

Antibodies used	HIF-1α, NB100-134; Novus biologicals<br>HIF-2α, NB100-122; Novus biologicals<br>Lamin A/C, 2032S; Cell signaling<br>OCN, AB10911; Merck<br>β-Actin, A3854; Sigma-Aldrich<br>RANKL, sc-7627; Santa cruz<br>Rabbit IgG, PP64B; EMD millipore<br>Goat anti-rabbit IgG peroxidase conjugate, A6154; Sigma-Aldrich
Validation	All antibodies are available on the supplier's website.

Eukaryotic cell lines

Policy information about cell lines and Sex and Gender in Research

Cell line source(s)	Calvarial pre-osteoblasts isolate from 3-days-old pups of ICR mouse.
Authentication	N/A
Mycoplasma contamination	N/A
Commonly misidentified lines<br>(See ICLAC register)	N/A

Animals and other research organisms

Policy information about studies involving animals; ARRIVE guidelines recommended for reporting animal research, and Sex and Gender in Research

Laboratory animals	Hif1afl/fl mice were obtained from Jackson Laboratory (#007561, Sacramento, CA, USA), and Col1a1-Cre mice were donated by Dr. Je-Yong Choi (Kyungpook National University, Daegu, Korea). To establish osteoblast-specific Hif1a knockout mice, Hif1afl/fl mice were backcrossed against Col1a1-Cre mice.
Wild animals	N/A
Reporting on sex	Male (4-month-old and 12-month-old), Female (ovariectomized)
Field-collected samples	All mice were fed and observed under laboratory standard conditions.
Ethics oversight	All procedures for animal care were approved by the Institutional Animal Care and Use Committee (IACUC) of Chonnam National University.

Note that full information on the approval of the study protocol must also be provided in the manuscript.

Plants

Seed stocks	N/A
Novel plant genotypes	N/A
Authentication	N/A
